# Metabolic Syndrome and Associated Factors in Adults of the Amazon Region

**DOI:** 10.1371/journal.pone.0167320

**Published:** 2016-12-09

**Authors:** Sérgio Lobato França, Sandra Souza Lima, José Ricardo Dos Santos Vieira

**Affiliations:** Institute of Biological Sciences of the Federal University of Pará, Belém, State of Pará, Brazil; State University of Rio de Janeiro, BRAZIL

## Abstract

Metabolic syndrome (MS) plays a key role in the origin of cardiovascular diseases. Studies on the MS in Brazil are recent, and its epidemiology in more isolated regions such as the Amazon is still unknown. The study aimed to estimate the prevalence of MS and associated factors in adults of the Brazilian Amazon. This study was conducted in 2012–2013. It is a cross-sectional population-based study, involving 787 adults randomly selected from the urban area of four cities in the state of Pará, in the Brazilian Eastern Amazon. The participants underwent anthropometric measurements, laboratory examination, and were questioned about their lifestyle. MS was defined by the Joint Interim Statement criteria, using the multiple logistic regression to investigate the potential association of risk factors with the presence of MS. The overall prevalence of MS was 34.1% (95% CI = 30.8–37.4), increasing linearly with the increasing body mass index and age. From 40–49 years of age, MS was observed in about half of the women (46.0%), while men only experienced a high prevalence in the fifth decade of life (43.3%). The low HDL-c (64.4%) and abdominal obesity (58.9%) were higher in women (p < 0.001), while for men, high blood pressure was significantly higher (p < 0.001). Individuals aged 40–59 years old (odds ratio [OR] = 3.35 [95% CI = 2.30–4.90]), ≥ 60 years old (OR = 5.80 [3.63–9.27]), overweight (OR = 4.17 [2.77–6.29]), and obese (OR = 8.82 [5.56–13.98]) were more likely to have MS. The study population experienced high cardiometabolic risk, requiring government efforts to control MS and related risk factors, especially obesity.

## Introduction

Metabolic syndrome (MS) is included in the list of non-communicable diseases, defined as an interconnected set of metabolic disorders that increase the risk for cardiovascular diseases (CVDs) and the chances of early mortality. Its main components are insulin resistance, high blood pressure, central obesity, and dyslipidemia (elevated triglycerides and low levels of high-density lipoprotein cholesterol) [1–3]. It can progress to cancer, gout, non-alcoholic fatty liver disease, polycystic ovary syndrome, sleep apnea syndrome, dementia, and other clinical complications [4]. Currently, CVDs are the leading causes of death in the world, especially ischemic heart disease and stroke, which collectively led to the death of 12.9 million people in 2010, equivalent to one in four deaths worldwide [5]. In Brazil thousands of hospitalizations occur annually due to coronary artery disease; only in 2011, it resulted in an estimated expense of about US$ 2.3 billion by the Unified Health System [6], denoting high impact on the country’s economy.

MS is already one of the most important complex disorders of the early 21st century, and is considered by some to be a global epidemic [2,7]. Currently, the MS condition occurs in one third of North-Americans [8], and already affects about 20% of the adult population in Europe [4]. In Latin America, it is estimated that MS affects approximately 24.9% of the population [9]. In Brazil, a major survey portrayed the prevalence in the adult population similar to that of developed countries, with an estimated average of 29.6% [10].

Since the recognition of MS as a clinical entity, its etiology and classification have been the subject of several discussions and modifications among various organizations and researchers because it is a set of complex diseases that involves all facets of CVD risk, whose pathophysiological and interactional mechanisms are still being clarified [3]. Currently a number of organizations and entities have set their criteria for classifying MS, among which the World Health Organization (WHO), National Cholesterol Education Program—Adult Treatment Panel III (NCEP-ATP III), American College of Clinical Endocrinology (AACE), European Group for the Study of Insulin Resistance (EGIR), and International Diabetes Federation (IDF) stand out. From 2009, in order to combine the various criteria, the National Heart, Lung, and Blood Institute—US (NHLBI), American Heart Association (AHA), World Heart Federation (WHF), International Atherosclerosis Society (IAS), International Association for the Study of Obesity (IASO), and IDF met to define a consensus on points of divergence for a better and global characterization of MS [1].

Studies on MS in Brazil are new, and in the Amazon, its epidemiological behavior is still unknown. Therefore, we sought to determine the prevalence of MS and associated factors according to sex, age, and the association of risk factors for MS in four cities of the State of Pará, in the Brazilian Eastern Amazon.

## Methods

### Ethical aspects

The project was approved by the ethics committee on human research of the Foundation Center of Hemotherapy and Hematology of Pará, State of Pará, Brazil (Report N° 0003.0.324.000–10) in accordance with the resolutions 347/2005 and 466/2012 of the National Council of Brazilian Health. We obtained written consent from participants, using a free informed consent form. All participants were offered access to their personal results in the study.

### Studied population and research design

**Fig 1:**
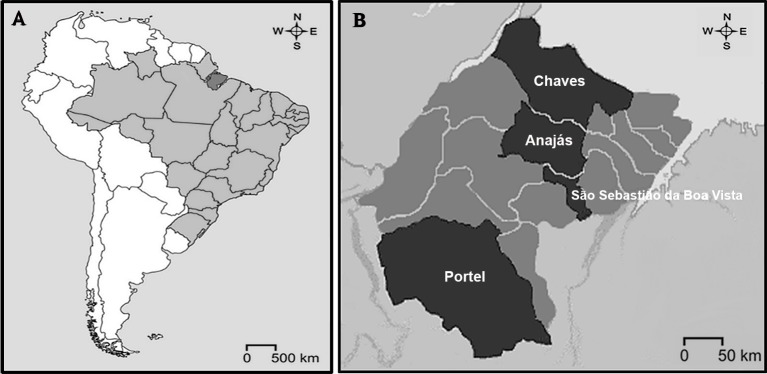
Location of the study area. A: Map of Brazil highlighting the Marajó region, State of Pará, Eastern Amazon; B: Marajó region highlighting the studied counties.

We conducted a cross-sectional population-based study involving adult participants (≥ 18 years old), and the study was a part of the project "Epidemiological Markers in Health in Marajó Archipelago", developed by the Federal University of Pará from September 2012 to April 2013, to assess the health of the resident population in the Marajó region, State of Pará, Eastern Amazon, Brazil (Fig 1). The research was conducted in the urban area of four strategic cities: São Sebastião da Boa Vista, Anajás, Portel, and Chaves. The Marajó region is located in the macro-region of the Northeast State of Pará, situated about 200 km distant from the capital, Belém, and consists of a large river system formed by the rivers Amazonas and Pará, comprising an area of 104,139.30 km^2^. It has a low population density with predominance of rural population over urban and access to best health establishments in capital is always difficult, mainly taking place by small fluvial boats, when travel often lasts about several days [11]. Its municipalities figure among the Brazilian cities with the lowest human development [12] and are included among the poorest regions of the country [13].

### Data collection

The sampling was random and stratified proportionally by municipalities, according to each population profile [14]. It was adopted a 90% confidence level, 1% sample error and a 1% minimum prevalence to a sample of 1,877 individuals: Chaves (n = 380), Anajás (n = 341), São Sebastião da Boa Vista (n = 359) and Portel (n = 797). Adding 5% taking into account possible losses, final sample was 1,964 individuals assisted throughout of the macro study that ranged from investigation of CVD risk to screening for cervical cancer, malaria and other diseases common in the region. Among these individuals, to describe prevalence of MS and associated factors, exclusion criteria were age ≤ 18 years old, pregnancy, refusal to provide blood sample, improper fasting, lack of anthropometric measurements, and incomplete biochemical examination. The final study sample contained 787 selected adults.

Participants were interviewed individually in a private place provided by the Basic Health Unit from municipalities, answering a questionnaire that covered demographic data and cardiometabolic risk factors, as well consumption of alcoholic beverages and smoking. For convenience, the samples were organized by sex and age groups (18–29 years old, 30–39 years old, 40–49 years old, 50–59 years old, and ≥ 60 years old). Data about food consumption and physical activity were not available because were part of another clinical approach that was not carried out by the field team.

### Diagnosis of metabolic syndrome

To diagnose MS, we used the criteria set by the joint scientific statement proposed by the NHLBI, AHA, WHF, IAS, IASO, and IDF in 2009 [1], which defines MS as the presence of three or more risk factors among five: 1) waist circumference ≥ 90 cm for men and ≥80 cm for women, according to reference for ethnic Central and South American population; 2) Triglycerides ≥ 150 mg/dL or drug treatment for this; 3) HDL-cholesterol < 40 mg/dL for men and < 50 mg/dL for women or drug treatment for this; 4) Blood pressure ≥ 130/85 mmHg or drug treatment for this; 5) Fasting glucose ≥ 100 mg/dL or drug treatment for this.

### Anthropometric evaluation

Anthropometric measurements included: a) blood pressure, measured manually using an aneroid sphygmomanometer and stethoscope (Prestige Medical, United States). The participants were properly positioned with the device in the left upper limb, sitting after a minimum interval of 5 min of rest. Three consecutive measurements were obtained, and the average of the last two measurements was used as the final result [15]; b) waist circumference (WC) measured with an inelastic tape measure (Mabis, United States) to the nearest 0.1 cm, measured from the midpoint between the iliac crests and the last ribs, with volunteers in an orthostatic position at the end of a respiratory expiration [16]; c) body weight, measured to the nearest 0.1 kg using a digital scale (model EKS 8897 REFLEX, France); d) height, measured by a portable stadiometer (WCS, Brazil) with an accuracy of up to 1 mm.

To classify the nutritional status of the participants, we used the body mass index (BMI) as follows: underweight (< 18.5 kg/m^2^); eutrophic (18.5–24.9 kg/m^2^); overweight (25.0–29.9 kg/m^2^); and obese (≥ 30 kg/m^2^), according to the criteria established by the World Health Organization [17].

### Biochemical evaluation

After fasting overnight for 12 h, blood samples from subjects were collected by venipuncture, to perform the serum biochemical evaluation of glucose, total cholesterol (TC), high-density lipoprotein cholesterol (HDL-c), and triglycerides. These measurements were performed in the field laboratory, using the Bio-2000 plus analyzer (Bioplus Ltda, Brazil). The low-density lipoprotein cholesterol (LDL-c) and very low-density lipoproteins (VLDL) cholesterol were calculated using the Friedewald equation [18], only in those samples with serum levels of triglycerides below 400 mg/dL. The non-HDL-C was obtained by the difference between TC and HDL-c.

### Risk factors

The risk factors assessed in participants were smoking and consuming alcoholic beverages. To characterize smoking, we considered three groups: non-smoking, those who never smoked or who had smoked for less than 6 months in a lifetime; smokers, those who smoked ≥ 1 cigarette/day for at least 6 months continuously; and former smokers, those who smoked regularly for more than 6 months during his lifetime, but they were not smokers at the time of the interview [19]. For assessing the habit of drinking alcohol, we considered: non-alcoholic, those who did not consume any alcohol or who consumed less than one serving per month; alcoholic, those who consumed ≥ 1 dose of any alcoholic beverage monthly; former alcoholic, those who declared to be alcoholic but they were not consumed alcohol at least one year before the interview [20].

### Data analysis

The sample size was calculated by BioEstat, version 5.3 [21], based on the calculation of the sample size for proportions, using a confidence interval of 90% and 0.1% significance level. Laboratory data and epidemiological questionnaire were coded and entered on a pre-programmed structure in Epi Info version 6.04 [22]. The continuous variables were presented as mean values ± standard deviation (SD); categorical variables were presented as absolute number, frequencies, and confidence interval (CI) of 95%. To evaluate the statistical differences between cities, sexes, and components of MS, we used the chi-square test, with the aid of BioEstat 5.3, at a significance level of 5% (p < 0.05). A multiple logistic regression was used to investigate the potential association between independent variables and the presence of MS (dependent), for which the criteria set for the inclusion of variables in the model was the association with the dependent variable in the univariate analysis (p < 0.20), ordered from the highest to the lowest significance. The interpretation of the model was carried out in accordance with the *p* value and odds ratio (OR) adjusted with CI. To carry out this analysis, we used Minitab Release 14 for Windows (Minitab Inc., State College, Pensilvânia, USA). The graphics were made with the aid of GraphPad Prism 5.0 (San Diego, California, USA).

## Results

### General characteristics of the population

The study involved 787 adults with an average age of 42.2 ± 16.3 years, with a predominance of female patients (76.1%). The prevalence of alcohol consumption and smoking were 25.4% and 12.2%, respectively. Regarding the nutritional status, there was a predominance of eutrophic (40.8%), while overweight occurred in 35.1% of the population and obesity in 22.0%. The mean BMI and WC were 26.8 ± 5.2 kg/m^2^ and 84.9 ± 12.2 cm, respectively. The mean systolic blood pressure was 129.1 ± 23.8 mmHg and the mean diastolic was 81.2 ± 16.5 mmHg. The mean serum levels obtained were the following: blood glucose = 91.9 ± 26.5 mg/dL; triglycerides = 112.0 ± 65.1 mg/dL; TC = 183.5 ± 44.8 mg/dL; HDL-c = 46.5 ± 11.8 mg/dL; LDL-c = 114.7 ± 40.6 mg/dL; VLDL-c = 22.4 ± 13.0 mg/dL, and non-HDL-c = 137.1 ± 45.7 mg/dL. Table 1 shows the other descriptive characteristics of participants, according to their city.

**Table 1:** General characteristics of the population. State of Pará, Eastern Amazon, Brazil, 2012–2013.

	Anajás (n = 175)	Chaves (n = 208)	Portel (n = 298)	SSBV (n = 106)	Total (n = 787)
Variables	n (%)	n (%)	n (%)	n (%)	n (%)
**Sex**					
Men	45 (25.7)	50 (24.0)	65 (21.8)	28 (26.4)	188 (23.9)
Women	130 (74.3)	158 (76.0)	233 (78.2)	78 (73.6)	599 (76.1)
**Age group**					
18–29 years old	55 (31.4)	54 (26.0)	72 (24.2)	25 (23.6)	206 (26.2)
30–39 years old	36 (20.6)	44 (21.2)	84 (28.2)	30 (28.3)	194 (24.7)
40–49 years old	34 (19.4)	41 (19.7)	42 (14.1)	20 (18.9)	137 (17.4)
50–59 years old	25 (14.3)	36 (17.3)	47 (15.8)	11 (10.4)	119 (15.1)
≥ 60 years old	25 (14.3)	33 (15.9)	53 (17.8)	20 (18.9)	131 (16.6)
**Smoking habits**					
Never smoked	98 (56.0)	132 (63.5)	162 (54.4)	63 (59.4)	455 (57.8)
Smoker	27 (15.4)	24 (11.5)	29 (9.7)	16 (15.1)	96 (12.2)
Former smoker	50 (28.6)	52 (25.0)	107 (35.9)	27 (25.5)	236 (30.0)
**Alcohol consumption**					
Non alcoholic	78 (44.6)	83 (39.9)	134 (45.0)	62 (58.5)	357 (45.4)
Alcoholic	49 (28.0)	61 (29.3)	69 (23.2)	21 (19.8)	200 (25.4)
Former alcoholic	48 (27.4)	64 (30.8)	95 (31.9)	23 (21.7)	230 (29.2)
**Nutritional status**					
Underweight (<18.5 kg/m^2^)	3 (1.7)	3 (1.4)	10 (3.4)	1 (0.9)	17 (2.2)
Eutrophic (18.5–24.9 kg/m^2^)	92 (52.6)	84 (40.4)	113 (37.9)	32 (30.2)	321 (40.8)
Overweight (25–29.9 kg/m^2^)	51 (29.1)	68 (32.7)	114 (38.3)	43 (40.6)	276 (35.1)
Obese (≥30 kg/m^2^)	29 (16.6)	53 (25.5)	61 (20.5)	30 (28.3)	173 (22.0)
**Anthropometric characteristics**	**Mean** ± **SD**	**Mean** ± **SD**	**Mean** ± **SD**	**Mean** ± **SD**	**Mean** ± **SD**
BMI (Kg/m^2^)	25.8 ± 4.7	27.6 ± 5.7	26.4 ± 5.0	27.9 ± 5.4	26.8 ± 5.2
WC (cm)	83.4 ± 11.7	84.7 ± 13.3	85.6 ± 12.3	85.9 ± 10.3	84.9 ± 12.2
ASP (mmHg)	127.7 ± 23.7	129.1 ± 22.8	128.6 ± 24.9	132.8 ± 22.6	129.1 ± 23.8
ADP (mmHg)	80.1 ± 15.7	81.3 ± 19.4	81.8 ± 16.1	81.2 ± 11.6	81.2 ± 16.5
**Biochemical characteristics**					
Fasting glycemia (mg/dL)	84.1 ± 13.5	93.7 ± 31.0	92.3 ± 24.1	99.9 ± 35.1	91.9 ± 26.5
Total Cholesterol (mg/dL)	184.4 ± 39.0	209.6 ± 46.1	166.5 ± 38.9	179.0 ± 42.8	183.5 ± 44.8
LDL-Cholesterol (mg/dL)	118.1 ± 36.4	141.7 ± 42.1	97.3 ± 30.2	105.0 ± 40.8	114.7 ± 40.6
VLDL-Cholesterol (mg/dL)	19.9 ± 10.8	26.4 ± 15.8	20.0 ± 11.0	25.5 ± 13.0	22.4 ± 13.0
HDL-Cholesterol (mg/dL)	46.5 ± 10.7	41.5 ± 11.1	49.2 ± 12.1	48.5 ± 11.3	46.5 ± 11.8
Cholesterol non-HDL (mg/dL)	137.9 ± 39.8	168.1 ± 47.6	117.3 ± 34.7	130.5 ± 45.5	137.1 ± 45.7
Triglycerides (mg/dL)	99.3 ± 54.0	131.8 ± 79.2	100.2 ± 54.9	126.9 ± 65.8	112.0 ± 65.1

SSBV: São Sebastião da Boa Vista; BMI: body mass index; WC: waist circumference; ASP: arterial systolic pressure; ADP: arterial diastolic pressure; LDL: low-density lipoprotein; VLDL: very low-density lipoprotein; HDL: high-density lipoprotein

### Prevalence of metabolic syndrome and its components

The overall prevalence of MS in the population was 34.1% (95% CI 30.8–37.4). For women, the prevalence was 35.4% (95% CI 31.7–39.3) and for men, 29.8% (95% CI 23.7–36.7), with no significant sex difference (p = 0.1846).

**Fig 2:**
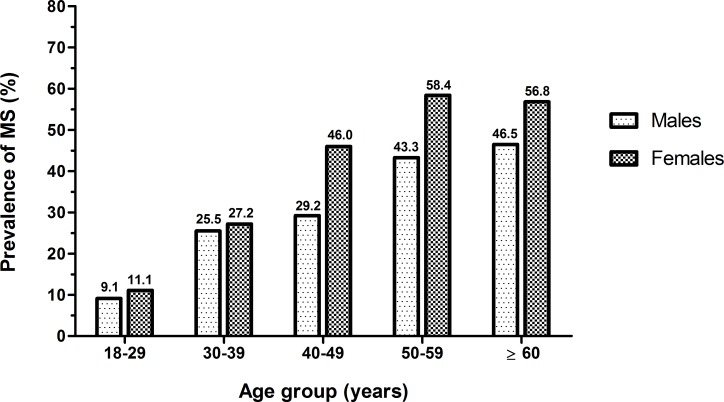
Distribution of the prevalence of metabolic syndrome according to age and sex. **State of Pará State, Eastern Amazon, Brazil, 2012–2013.** MS = Metabolic syndrome.

Among the cities, the distribution of MS occurred heterogeneously (p < 0.001); SSBV was the city with the highest prevalence (46.2%), followed by Chaves (44.2%), Portel (28.5%), and Anajás (24.0%). Through age stratification, we noted that in both sexes, the frequency of MS increased with advancing age. For women, the occurrence of MS reaches high prevalence after 40 years (46.0%), while among men, MS was more evident from 50 years of age (43.3%), as shown in Fig 2.

**Fig 3:**
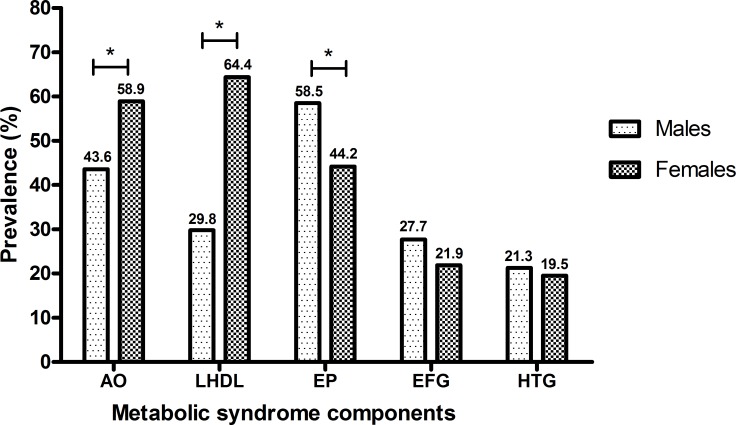
Distribution of the prevalence of metabolic syndrome components according to sex. **Pará State, Eastern Amazon, Brazil, 2012–2013.** AO = abdominal obesity; LHDL = low levels of high density lipoprotein cholesterol; EP = elevated pressure; EFG = elevated fasting blood glucose; HTG = hypertriglyceridemia. *P value < 0.05 (chi-square test).

The most common components of MS in the general population of the municipalities were low HDL-c (56.2%), abdominal obesity (55.3%), and high blood pressure (47.6%), and elevated fasting blood glucose (24.3%). The hypertriglyceridemia was the least frequent component (19.9%). It was observed that MS components varied according to sex. For women, low HDL-c (64.4%) and abdominal obesity (58.9%) were significantly higher than for men (p < 0.001). Meanwhile, high blood pressure for men (58.5%) was significantly higher (p < 0.001) that that for women (Fig 3).

### Association of risk factors for metabolic syndrome

The risk factors that correlate with MS by univariate analysis were individuals of 40–59 years of age (OR = 2.52, 95% CI = 1.85–3.45, p < 0.001), ≥ 60 years (OR = 2.65, 95% CI = 1.81–3.89, p < 0.001), former smokers (OR = 1.68, 95% CI = 1.22–2.30, p = 0.001), overweight (OR = 1.93, 95% CI = 1.42–2.62, p < 0.001), and obese (OR = 3.64, 95% CI = 2.56–5.16, p < 0.001) (Table 2).

**Table 2:** Univariate analysis of risk factors related to the presence of metabolic syndrome. State of Pará, Eastern Amazon, Brazil, 2012–2013.

Variables		Total (n = 787)	Presence of MS n (%)	Odds Ratio (95%CI)	p value
***Sex***					
	Men	188	56 (29.8)	1.0	
	Women	599	212 (35.4)	0.77 (0.54–1.10)	0.158
***Age group***					
	18–39 years old	400	74 (18.5)	1.0	
	40–59 years old	256	124 (48.4)	2.52 (1.85–3.45)	<0.001
	≥ 60 years old	131	70 (53,4)	2.65 (1.81–3.89)	<0.001
***Smoking habits***					
	Never smoked	455	137 (30.1)	1.0	
	Smoker	96	31 (32.3)	0.91 (0.58–1.44)	0.698
	Former smoker	236	100 (42.4)	1.68 (1.22–2.30)	0.001
***Alcohol consumption***					
	Non alcoholic	357	129 (36.1)	1.0	
	Alcoholic	200	61 (30.5)	0.81 (0.57–1.14)	0.220
	Former alcoholic	230	78 (33.9)	0.99 (0.72–1.37)	0.957
Nutritional status*					
	Eutrophic (18.5–24.9 kg/m^2^)	321	45 (14.1)	1.0	
	Overweight (25–29.9 kg/m^2^)	276	121 (43.8)	1.93 (1.42–2.62)	<0.001
	Obese (≥ 30 kg/m^2^)	173	100 (57.8)	3.64 (2.56–5.16)	<0.001

MS: metabolic syndrome; 95% CI: confidence interval of 95%.

*The quantitative difference is due to the exclusion of malnourished individuals.

In the final model of multivariate regression, after adjustment with all the other variables, only middle-aged (40–59 years old), elderly (≥ 60 years old), overweight, and obese subjects maintained statistical significance. This demonstrated that overall, obesity was the most related risk factor for MS, with nine times the approximate risk of occurrence of MS (OR = 8.82, 95% CI = 5.56–13.98, p < 0.001) (Table 3).

**Table 3:** Logistic regression model of risk factors associated with metabolic syndrome. State of Pará, Eastern Amazon, Brazil, 2012–2013.

Variables	Odds Ratio Adjusted	(95%CI)	p value
Age: 40–59 years old	3.35	2.30–4.90	<0.001
Age: ≥ 60 years old	5.80	3.63–9.27	<0.001
Overweight (25–29.9 kg/m^2^)	4.17	2.77–6.29	<0.001
Obese (BMI ≥ 30 kg/m^2^)	8.82	5.56–13.98	<0.001

BMI: body mass index; 95% CI: confidence interval of 95%.

## Discussion

The overall prevalence of MS observed in our results (34.1%) was considerably higher than the frequency reported in the Latin American population [9, 23] and greater than the estimated prevalence for the Brazilian population (29.6%) [10], exceeding for example, the prevalence in São Paulo (22.7%) [24] and Brasília (32.0%) [25], which are major urban centers in the country and evaluated by the same criteria for diagnosis.

We observed that this syndrome affects women earlier than men, with a significant increase in the age group of 40–49 years, since nearly half the women in this age had been affected (46%), and men only reached high prevalence from the fifth decade of life (43%). This result differed from those observed in other studies [10, 24], which have only shown high prevalence of MS in individuals over 50 years old. Therefore, it is necessary to better understand the various aspects involving this early propensity for MS in women, such as, for example, the correlation with the average age of menopause. There have been indications of early menopausal signs in women from other Amazonian cities [26], and when menopause occurs, it is responsible for triggering negative changes in the cardiometabolic homeostasis of women, which promotes obesity, dyslipidemia, and other risk factors for MS, given the loss of cardiovascular protection afforded by ovarian hormones prior to menopause [27, 28].

There was a significant difference between the assessed municipalities, and the cities of SSBV and Chaves had the highest frequencies of MS in their populations with 46.2% and 44.2%, respectively. This explains the significant increase in obesity in these municipalities, and consequently, a greater risk for onset of MS [29–32].

In our results, diabetes mellitus (DM) prevalence could not be estimated because fast blood glucose above ≥ 100mg/dL was adopted as criterion for MS and was not available clinical diagnosis or other parameters as well as insulin and glycated hemoglobin due the field laboratory carried out only basic tests, and the high cost of these tests in diagnostic centers in the capital was out of project budget. Despite technical limitations, the percentage of adults with elevated fasting blood glucose (24.3%) was far higher the prevalence of DM in 2014 of 9.0% in men and 7.9% in women estimated by WHO in meta-analysis of 751 studies carried out over 35 years of analysis, including 4,372,000 adults from 146 countries [33]. In comparison with this same study, our results were close to the prevalence of Polynesia and Micronesia (25%), but were threateningly higher than the percentage of adults who reported a medical diagnosis of diabetes (7.6%) estimated for the Pará capital in comprehensive study carried out in Brazil in 2014 [34].

The most common components of MS described in this study (low HDL-c, abdominal obesity, and high blood pressure) corroborated with what was described in previous studies [9, 10, 35, 36]. We observed that the MS components varied according to sex, and the low HDL-c component was the most frequent in women, since they show twice the changes in this component as compared to men (64.4% versus 29.8%). There is evidence that low levels of HDL-c in the female population can be a common finding in the Brazilian population [10, 36] and Latin American populations [9]. However, low HDL-c is an independent risk factor for MS, because when its levels are suitable it is able to remove cellular cholesterol, in addition to playing important antioxidant and antithrombotic roles, which improves endothelial function and inhibits atherosclerosis [37–39].

High blood pressure was the most important component for men, affecting half of the male population, and statistically higher than in women (58.5% versus 44.2%). This disparity between sexes has also been described in other regional studies [40–42].

Hypertension, characterized by high prevalence and low rates of blood pressure control, is an important risk factor for CVD mortality. In the Brazilian population, the percentage of adults who reported a medical diagnosis of hypertension is estimated around 24.8%, higher in women (26.8%) than men (22.5%); in the capital of Pará the percentage of hypertensive adults is lower (19.1%), with 15.8% of men and 18.7% women [34]. it is estimated that around 30% of adults are hypertensive [15]; In our study, despite the cutoff value reaching the limit for high blood pressure (≥ 130/85 mmHg), the high prevalence increase in men is alarming. Currently, the population of African descent people in the State of Pará corresponds to 77.7% [43], and in Marajó, the miscegenation between poor whites, blacks, and Indians is historically a natural process [44]. Thus, the ethnogenetics of this population may have favored the prevalence found in these places because heredity plays a role in the genesis of hypertension, since hypertension in Afro-descendant individuals is twice as common [15].

When risk factors were adjusted with all the other variables, we found that middle-aged, elderly, overweight, and obese individuals were significantly more likely to suffer from MS. Overweight individuals showed 4 times higher risk in developing MS, and for obese individuals, this risk further doubled (OR: 8.82). Accordingly, such proportionality was also observed for age, where individuals from 60 years of age showed an approximately doubled risk (OR: 5.8) to develop MS compared to the group of 40–59 year old participants (OR: 3.3). Ratified along with other studies, age and obesity are the most significant predictors of MS [9, 10, 25, 45]. Increased adiposity, regardless of age, increases the risk of MS by adding a high risk for many chronic diseases such as type 2 diabetes, hypertension, fatty liver, dyslipidemia, cancer, and CVD, among others [29, 46–48]. The high prevalence of obesity found in our sample is consistent with the rapid advance of obesity that is observed throughout the country. In an obesity worldwide mapping, Brazil stood out among the countries that have experienced greater increase in the absolute number of overweight and obese people between 1980 and 2008, behind only China and the United States [49]. Currently, about 18% of the Brazilian adult population is obese, and half is overweight (52.5%) [34]. Ours results demonstrate that Marajó Archipelago already exceed the global obesity prevalence projected for 2025 by a comprehensive prospective study carried out by WHO about non-communicable diseases with more than 19.2 million adult participants in 186 countries from 1975 to 2014, that estimates the obesity prevalence based in BMI will reach 18% in men and surpass 21% in women [50].

The recent geometric growth of MS in developing countries has been attributed mostly to increased industrialization favoring the increase of imported technology and cultural adaptations. This has a negative effect on eating habit changes favoring an increase in sedentary lifestyle in the population [51–53]. The high prevalence of MS found in the studied cities are evidence of this transition, especially in relation to food habits, as increasing urbanization promotes greater availability of processed foods, which are easily available and diverse, thus becoming a part of the daily lives of these people. An example is the frozen chicken from the South and Southeast of the country, arriving to communities transported by boats, replacing the traditional diet based on fish and manioc flour [54,55]. The same happens with soft drinks, which are cheap and even more accessible than local food with açaí, a traditional drink of these people with great nutritional value, especially important for its anti-inflammatory effects and beneficial immune profile in dyslipidemia, type 2 diabetes, MS, cancer, and aging [56,57]. This progress perspective, which indirectly encourages an increasingly westernized lifestyle, undermining the local sociocultural provisions that gave food security to these people in the Amazon. This causes an increase in energy density in the meals, compromising the self-regulation of metabolic balance, and thus, increasing the risk of obesity and MS in this population [58].

It is important to highlight that MS has been associated independently with low socioeconomic status and low education levels [59–61]. Therefore, the studied cities have an aggravating factor by being in one of the poorest regions of Brazil, with great need for infrastructure development, higher education, proper housing, and greater presence of the State [13]. These factors can contribute to the intensification of MS in this region, given that individuals who have financial stability and higher education are less likely to have chronic diseases as they have better access to information, healthy food, and better health level assistance [33, 61].

In this study, the fewer number of men compared to women may have compromised the comparative analysis between sexes. However, it has been reported that commonly, men have a low demand for health services, where the social imagery considers man as invulnerable and contributes to them to look less for health services [62]. It should be recorded that currently there is no WC cutoffs for the Brazilian population, and when using the general pattern for the population of South America, which is recommended [1], a value is imposed that may not reflect the phenotype most suitable of the Brazilian population, given that some studies have shown a distinct pattern [63, 64]. The cross-sectional design of the study naturally has a low ability to demonstrate causality compared to longitudinal studies, and in some respects, constitutes a barrier. Despite these limitations, our work is important to unprecedentedly describe MS and cardiovascular risk factors in cities of the Brazilian Amazon, and the data of this very important study can be used to better understand the epidemiology of MS in Brazil and worldwide.

## Conclusions

The prevalence of MS in these Amazonian cities was higher than that observed in other Brazilian regions, and its frequency increased linearly with increasing BMI and age. In order to reduce the risk of morbidity and mortality from CVD, the prevention and treatment of MS should be priority for the local public health agencies. For ongoing and future studies, since the factors triggering the MS condition are of complex origin, the ethnogenetic composition, food consumption and socioeconomic conditions are important points to consider.

Further work must be developed using data from other projects of our work team that collected data from socioeconomic status, nutrition conditions, physical activity and other possible factors related to MS.

Our research group has organized health education actions in the municipalities by presenting results of this study and a report was presented to authorities in public health of Pará State and municipalities from Marajó archipelago in order to orientate immediate actions and to establish efficient policies to reverse or even taking over control responsibility to plan measures for decreasing risk of non-communicable diseases.
